# Close correlation between thiolate basicity and certain NMR parameters in cysteine and cystine microspecies

**DOI:** 10.1371/journal.pone.0264866

**Published:** 2022-03-11

**Authors:** Juliana Ferreira de Santana, Arash Mirzahosseini, Beáta Mándity, Dóra Bogdán, István Mándity, Béla Noszál

**Affiliations:** 1 Department of Pharmaceutical Chemistry, Semmelweis University, Budapest, Hungary; 2 Research Group of Drugs of Abuse and Doping Agents, Hungarian Academy of Sciences, Budapest, Hungary; 3 MTA TTK Lendület Artificial Transporter Research Group, Institute of Materials and Environmental Chemistry, Research Center for Natural Sciences, Hungarian Academy of Sciences, Budapest, Hungary; 4 Department of Organic Chemistry, Semmelweis University, Budapest, Hungary; The University of Texas at El Paso, UNITED STATES

## Abstract

The imbalance between prooxidants and antioxidants in biological systems, known as oxidative stress, can lead to a disruption of redox signaling by the reactive oxygen/nitrogen species and is related to severe diseases. The most vulnerable moiety targeted by oxidant species in the redox signaling pathways is the thiol (SH) group in the cysteine residues, especially in its deprotonated (S^−^) form. Cysteine, along with its oxidized, disulfide-containing form, cystine, constitute one of the most abundant low molecular weight biological redox couples, providing a significant contribution to the redox homeostasis in living systems. In this work, NMR spectra from cysteine, cystine, and cysteine-containing small peptides were thoroughly studied at the submolecular level, and through the chemical shift data set of their certain atoms it is possible to estimate either thiolate basicity or the also related standard redox potential. Regression analysis demonstrated a strong linear relationship for chemical shift vs thiolate log*K* of the cysteine microspecies data. The ^α^CH ^13^C chemical shift is the most promising estimator of the acid-base and redox character.

## 1. Introduction

The imbalance between prooxidants and antioxidant pathways in biological systems, known as oxidative stress, can lead to a disruption of redox signaling by the reactive oxygen/nitrogen species and is related to aging, atherosclerosis, carcinogenesis, diabetes, and neurodegeneration [1, 2]. Although the abovementioned reactive oxidizing species have some essential role against infectious pathogens and in cellular signaling systems, their effects are favorable only if they are present in low or moderate concentrations and under tight cellular regulation [3].

The primary chemical moiety targeted by oxidizing species in the redox signaling pathways is that of the thiol-containing cysteine (CysSH or Cys). Cysteine oxidation is notable in pathological mechanisms when an oxidized protein has its activity modified and is vulnerable to aggregation and degradation [4]. The most common form of oxidized cysteine prevalent in biochemical processes is cystine (CysSSCys or (Cys)_2_), which contains a disulfide bond; a result of oxidation by loss of two electrons from two cysteine species each bearing a thiolate group. The cysteine/cystine redox couple is one of the most abundant low molecular weight redox couples in human plasma, rivaled only by the tripeptide-type glutathione(GSH)/glutathione disulfide(GSSG) redox buffer [5]. In 2016, our research group introduced an indirect method using (species-specific) standard redox potentials to characterize thiolate-disulfide equilibria with pH-independent parameters [6]. This is necessary to thoroughly explore the oxidation process in which only the thiolate moiety participates, untangled from the parallel acid-base processes.

Despite being a pivotal regulator of redox homeostasis and signaling, not all cysteine residues present in proteins are likely to be oxidized; disulfide bridge formation will depend on a few other factors, such as solvent accessibility, p*K*_a_ (henceforth dealt in terms of protonation constant, log*K*), polarity of nearby residues, and steric proximity.

In order to better comprehend the biological function of cysteine oxidation and establish an antioxidant therapy, which could eliminate the currently unmet medical need of oxidative stress [7, 8], it is inevitable to find new ways to reveal the possible relationships between the subtle and co-dependent redox, acid-base and spectroscopic properties.

A complete microspeciation of cysteamine, cysteine, homocysteine, and their respective homodisulfides has been previously elaborated by means of ^1^H NMR-pH titrations allowing the comprehension of the detailed acid-base processes of thiol-containing amino acids at a submolecular level [9, 10]. Now we are extending the observed correlation between standard redox potentials and thiolate log*K* [6] to chemical shift values, to highlight the predictive power of NMR parameters available from relatively simple, single spectroscopic measurements.

## 2. Materials and methods

### 2.1 Materials

Cysteine and cystine were purchased from Sigma (Merck) and were used without further purification. Deionized water was prepared with a Milli-Q Direct 8 Millipore system. Compounds **1**–**11** were purchased from ProteoGenix (Schiltigheim, France).

The cysteine derivatives (**12**–**15**) were synthesized on TentaGel R RAM resin (0.19 mmol/g) with Fmoc-chemistry on a Rink amide linker on a 0.1 mmol scale manually. The coupling of cysteine was performed as follows: 3 equivalents of Fmoc-protected amino acid, 3 equivalents of the uronium coupling agent O-(7-azabenzotriazol-1-yl)-N,N,N′,N′-tetramethyluronium hexafluorophosphate (HATU) and 6 equivalents of N,N-diisopropylethylamine (DIPEA) were used in N,N-dimethylformamide (DMF) as a solvent with shaking for 3 h. After the coupling steps, the resin was washed 3 times with DMF, once with methanol and 3 times with dichloromethane. Deprotection was performed with 2% 1,8-diazabicyclo [5.4.0] undec-7-ene (DBU) and 2% piperidine in DMF in two steps, with reaction times of 5 and 15 min. After the deprotection of the Fmoc-group, the resin was washed and a further coupling step was carried out with the appropriate benzoic acid derivative and HATU with DIPEA as coupling agent. The resin was washed with the same solvents as described previously. The cleavage was performed with trifluoroacetic acid/water/DL-dithiothreitol (DTT)/triisopropylsilane (TIS) (90:5:2.5:2.5) at 0°C for 2 h. The cleavage cocktail is evaporated, and the peptide is precipitated with diethyl ether. After the precipitation, the cysteine derivatives are dissolved in 10% acetic acid solution and are lyophilized. As a final purification, the solid residue that remained after lyophilization is digerated with diisopropyl ether. The crystals gained by this step were washed with diisopropyl ether.

### 2.2 NMR spectroscopy measurements

NMR spectra were recorded on a Varian Unity Inova DDR spectrometer (599.9 MHz for ^1^H) with a 5 mm ^1^H{^13^C/^31^P-^15^N} pulse field gradient triple resonance probehead at 298.15 ± 0.1 K. The solvent was H_2_O:D_2_O 95:5 (*V*/*V*), ionic strength was adjusted to 0.15 mol/L with KCl. The pH values were adjusted with HCl or NaOH and determined *in situ* by internal indicator molecules (at ca. 1 mmol/L) optimized for ^1^H NMR [11, 12]. The sample volume was 550 μL and every sample contained ca. 1 mmol/L DSS (3-(trimethylsilyl) propane-1-sulfonate) as chemical shift reference. The H_2_O ^1^H signal was suppressed with a presaturation sequence; the average acquisition parameters for ^1^H measurements are: number of transients = 16, number of points = 65536, acquisition time = 3.33 s, relaxation delay = 1.5 s. ^1^H-^13^C HSQC measurements were performed with solvent signal presaturation and the following parameters: number of transients = 64, number of increments = 96, number of points = 2884, acquisition time = 149.968 ms, relaxation delay = 1 s.

### 2.3 Statistical analysis

Non-linear regression analyses on the titration data were carried out using R version 4.0.5 (R Foundation for Statistical Computing, Vienna, Austria) [13] with the following function:

$$\delta_{\text{obs}\;\left(\text{pH}\right)}=\frac{\delta_{\text{L}}+\delta_{\text{HL}}\times 10^{\text{log}K-\text{pH}}}{1+10^{\text{log}K-\text{pH}}}$$ (1)

where *δ*_L_ is the chemical shift of an unprotonated moiety, *δ*_HL_ is the chemical shift of the protonated moiety, and log*K* is the base 10 logarithm of the group-specific protonation constant. Linear regression analyses for the chemical shift-log*K* data were carried out using the R version 4.0.5 (R Foundation for Statistical Computing, Vienna, Austria) [13].

## 3. Results

### 3.1 Cysteine and cystine species-specific chemical shift data

**Fig 1:**
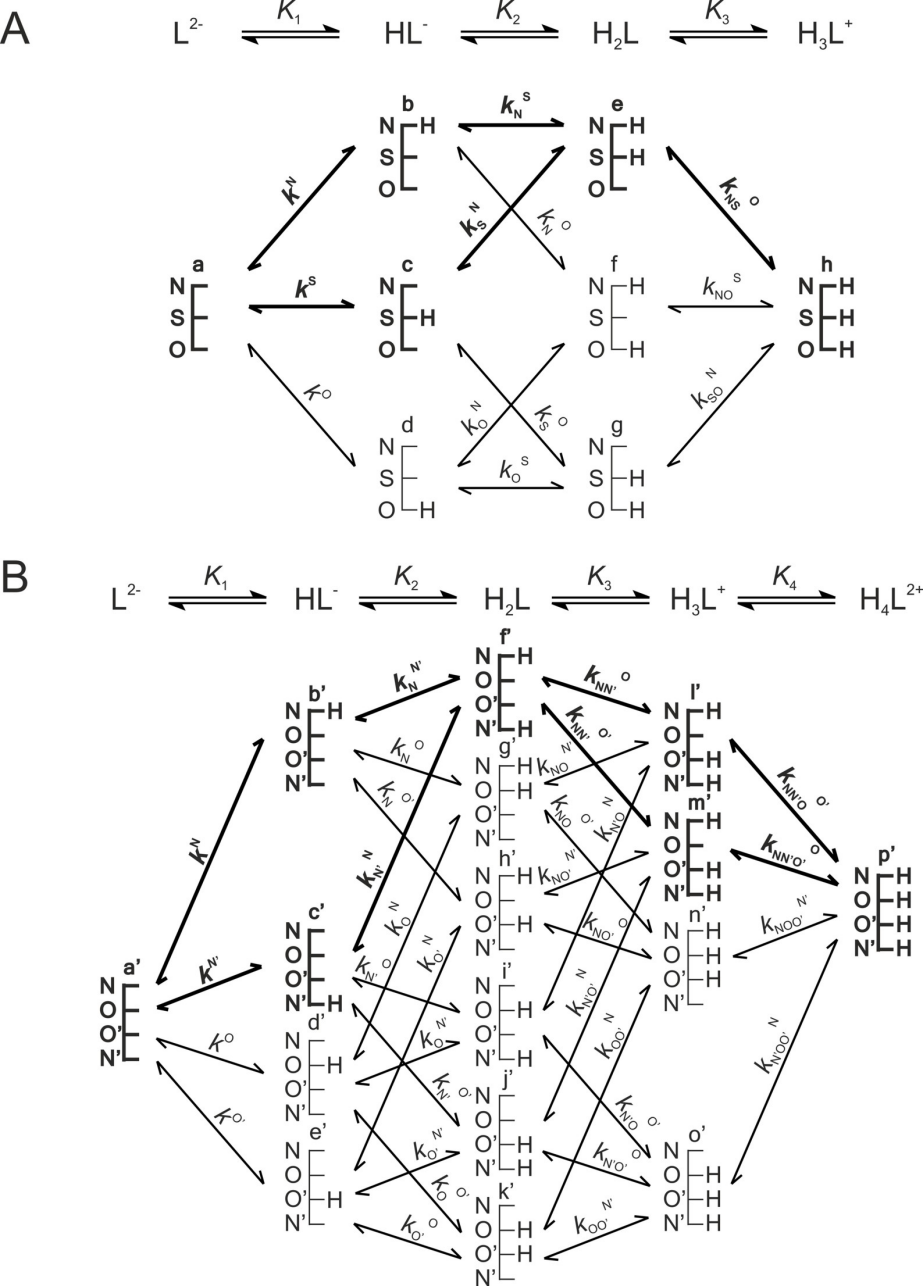
This figure was reproduced from [6, 10]. In this figure the protonation equilibrium schemes of cysteine Cys (A) and cystine (Cys)_2_ (B) are depicted. Stepwise macroscopic protonation constants (*K*_1_, *K*_2_, *K*_3_ …) characterize overall protonation state of the compounds, where L^2−^, HL^−^, etc. are the successively protonating ligands. In the schematic networks the species-specific protonation pathways are depicted in terms of microspecies (a, b, c …) and microscopic protonation constants (*k*^N^, *k*^N^_S_…) that characterize the submolecular localization of the protonation within the molecule. The components of the major pathways are in bold; these microspecies have orders of magnitude larger relative abundance among their protonation isomers. Symbols N, S, and O represent the amino, thiolate, and carboxylate basic moieties, respectively.

The acid-base microspeciation schemes–with the symbols of the various species–of cysteine and cystine are presented in Fig 1. The species-specific protonation constants of cysteine and cystine are identical with those in previous works [9, 14]. The species-specific NMR chemical shifts of the ^α^CH and ^β^CH_2_ nuclei were determined by measuring ^1^H and ^1^H-^13^C HSQC NMR spectra at limiting pH values (corresponding to the plateaus on the titration curves of the compounds, see S1 and S2 Figs). The species-specific chemical shifts of the cysteine and cystine microspecies were determined using Submeier-Reilley equations [15]; this method was recently elaborated for the analogous selenocysteine/selenocystine pair [16]. Briefly, first the chemical shifts were recorded at limiting pH values (i.e. at the plateaus of the titration curve of the compound); these chemical shifts afforded the species-specific chemical shift values of the major microspecies (see major microspeciation pathway in Figs 1 and 3), since the contribution of minor microspecies to the observed chemical shifts is insignificant. The chemical shifts of the major microspecies also afford the protonation shifts (Δ*δ*) associated with the various basic moieties, which in turn allow the determination of the NMR chemical shifts of the minor microspecies as well. The species-specific chemical shifts are compiled in Table 1 grouped according to thiolate-bearing, thiol-bearing, and the complementary disulfide-bearing microspecies, respectively. The thiolate-specific protonation shifts determined for cysteine on the ppm scale are as follows: Δ*δ*^1^H(^β^CH_2_) = 0.29 and −0.03; Δ*δ*^13^C(^β^CH_2_) = −2.0; Δ*δ*^1^H(^α^CH) = 0.36; Δ*δ*^13^C(^α^CH) = −2.6.

**Table 1:** The species-specific chemical shifts (on the ppm scale) determined for cysteine and cystine microspecies. The uncertainty of determination for chemical shift on the ppm scale is 0.001 and 0.01 for ^1^H and ^13^C chemical shift values, respectively. For the thiol- and disulfide-bearing species, the concomitant thiolate protonation constant given in the third column refer to the relevant thiolate-bearing microspecies that give rise to such thiol- or disulfide-bearing species via protonation or oxidation, respectively. The thiolate protonation constants were determined previously in [9].

class of species	microspecies	thiolate log*k*	^β^CH_2_			^α^CH	
			*δ*^1^H		*δ*^13^C	*δ*^1^H	*δ*^13^C
thiolate-bearing Cys	Cys a	10.07±0.03	2.460	2.894	34.75	3.099	63.13
	Cys b	8.76±0.04	2.746	3.141	29.61	3.631	61.25
	Cys d	8.95±0.01	2.566	2.984	33.87	3.479	61.72
	Cys f	7.64±0.01	2.853	3.231	28.73	4.011	59.84
thiol-bearing Cys	Cys c	10.07±0.03	2.745	2.861	32.74	3.461	60.54
	Cys e	8.76±0.04	3.032	3.108	27.60	3.993	58.66
	Cys g	8.95±0.01	2.852	2.951	31.86	3.841	59.13
	Cys h	7.64±0.01	3.138	3.198	26.72	4.373	57.25
disulfide-bearing (Cys)_2_	(Cys)_2_ a’	10.07±0.03	2.899	3.106	46.27	3.575	57.74
	(Cys)_2_ f’	8.76±0.04	3.186	3.387	40.23	4.125	56.05
	(Cys)_2_ k’	8.95±0.01	3.018	3.205	45.17	3.971	56.40
	(Cys)_2_ p’	7.64±0.01	3.335	3.478	38.76	4.526	54.61

**Fig 2:**
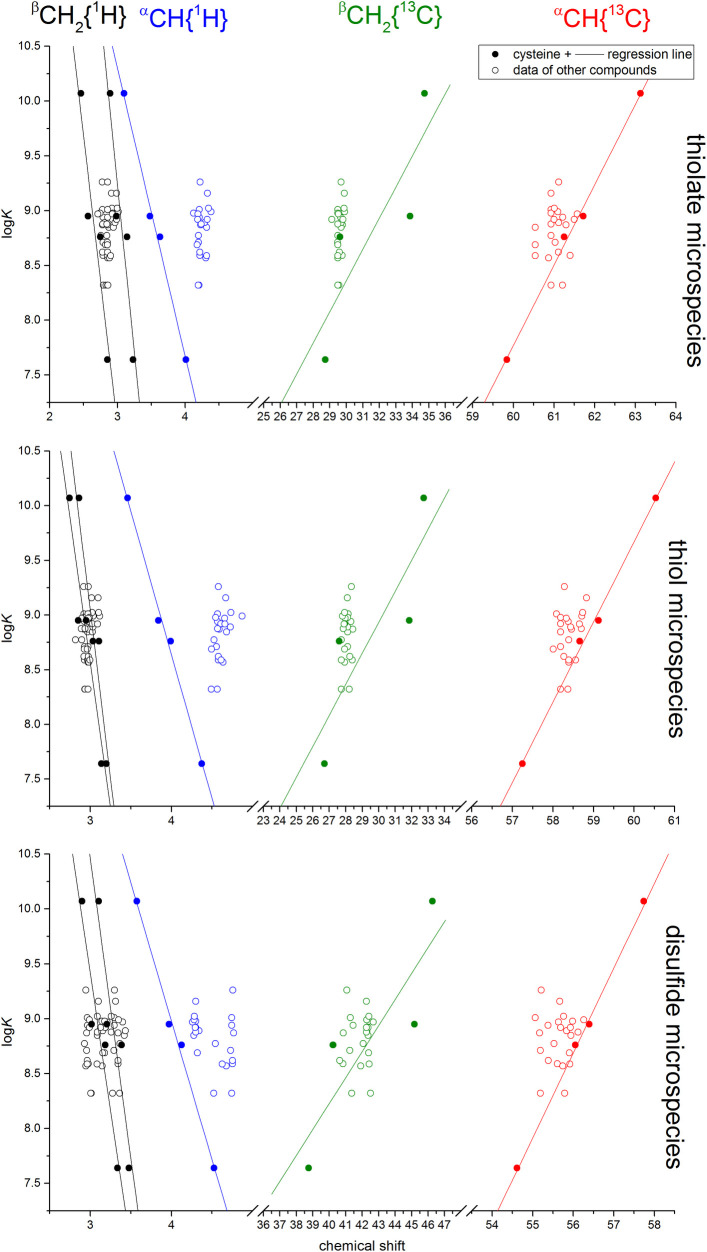
The multivariate linear regression fits of the chemical shift data of cysteine data (solid circles) vs cysteine species-specific thiolate basicities. The chemical shift data of the others (see section 3.2) are superimposed on this regression fit with empty circles.

We performed separate multiple linear regression analyses on the data found in Table 1 using the NMR chemical shifts as independent variables and the log*K* as dependent variable; this result is depicted in Fig 2 with solid scatter points and regression lines. Note that the assignment of independent and dependent variables is not meant to reflect causal relationship between the parameters, but is purely designed to establish a model to predict log*K* values from chemical shifts. The results showed that for each of the three cases the ^α^CH ^13^C chemical shift had the most reliable contribution to the model. It can also be seen from the scatter plots in Fig 2 that this chemical shift has the best fit and predictive potential on the log*K* values. The parameters of the multiple linear regression analysis are presented in Table 2. It is noteworthy to make certain distinctions between the regression parameters and their interpretation; (a) the adjusted R^2^ characterizes the vertical dispersion of the data points around the linear fit and quantifies how much the linear model explains the variability of the data; (b) the slope of the regression line characterizes the degree and direction of response between the dependent and independent variable, i.e. how much is a particular thiolate basicity accompanied by a different chemical shift; (c) thiolate-specific protonation shift is the chemical shift change a nucleus undergoes when the thiolate moiety changes protonation state from unprotonated (thiolate) to the protonated (thiol) form.

**Table 2:** The regression statistics of the cysteine and cystine chemical shift data.

		Intercept		Slope		Statistics	
		Value	Standard error	Value	Standard error	Adj. R-square	P-value
thiolate species	^β^CH_2_{^1^H}	22.92	3.65	-5.294	1.373	0.8222	0.0611
	^β^CH_2_{^1^H}	27.66	4.98	-6.141	1.624	0.8159	0.0634
	^β^CH_2_{^13^C}	-0.16	3.81	0.284	0.120	0.6064	0.1412
	^α^CH{^1^H}	18.19	0.58	-2.626	0.162	0.9886	0.0038
	^α^CH{^13^C}	-36.21	2.01	0.733	0.033	0.9941	0.0020
thiol species	^β^CH_2_{^1^H}	24.43	4.04	-5.294	1.373	0.8222	0.0611
	^β^CH_2_{^1^H}	27.46	4.93	-6.141	1.624	0.8159	0.0634
	^β^CH_2_{^13^C}	0.41	3.57	0.284	0.120	0.6064	0.1412
	^α^CH{^1^H}	19.14	0.64	-2.626	0.162	0.9886	0.0038
	^α^CH{^13^C}	-34.31	1.92	0.733	0.033	0.9941	0.0020
disulfide species	^β^CH_2_{^1^H}	24.38	3.33	-4.992	1.069	0.8739	0.0430
	^β^CH_2_{^1^H}	26.86	5.07	-5.465	1.537	0.7952	0.0708
	^β^CH_2_{^13^C}	-1.25	3.96	0.237	0.093	0.6492	0.1247
	^α^CH{^1^H}	19.07	0.60	-2.522	0.148	0.9897	0.0034
	^α^CH{^13^C}	-34.60	1.20	0.773	0.021	0.9977	0.0008

### 3.2 Species-specific chemical shift data of cysteine-containing peptides

**Fig 3:**
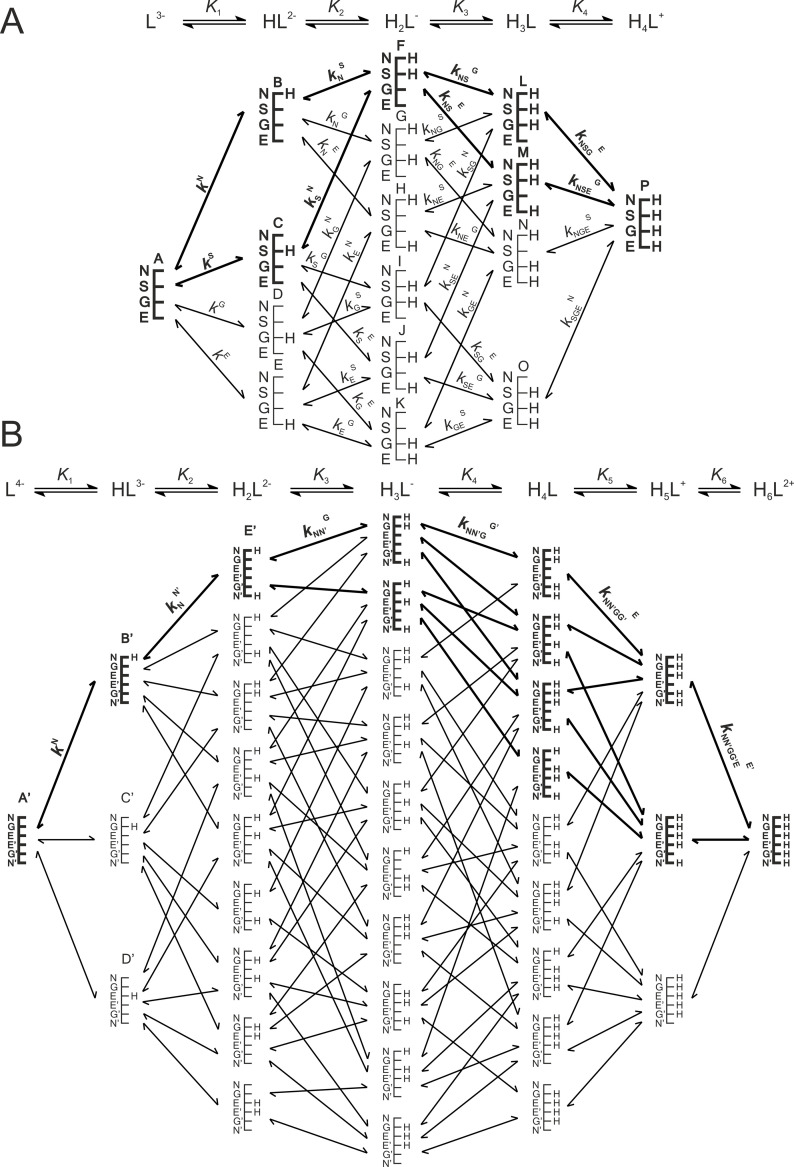
This figure was reproduced from [6, 10]. The protonation macro- and microequilibrium schemes of glutathione GSH (A) and glutathione disulfide GSSG (B) are presented in a similar fashion to Fig 1. For the symmetrical glutathione disulfide only non-identical microspecies and microconstants are shown, and only a few of the microconstants are labeled. The components of the major pathways are in bold. The basic moieties are depicted with their one-letter symbols: N (amino), S (thiolate), G (glycyl carboxylate), and E (glutamyl carboxylate).

**Fig 4:**
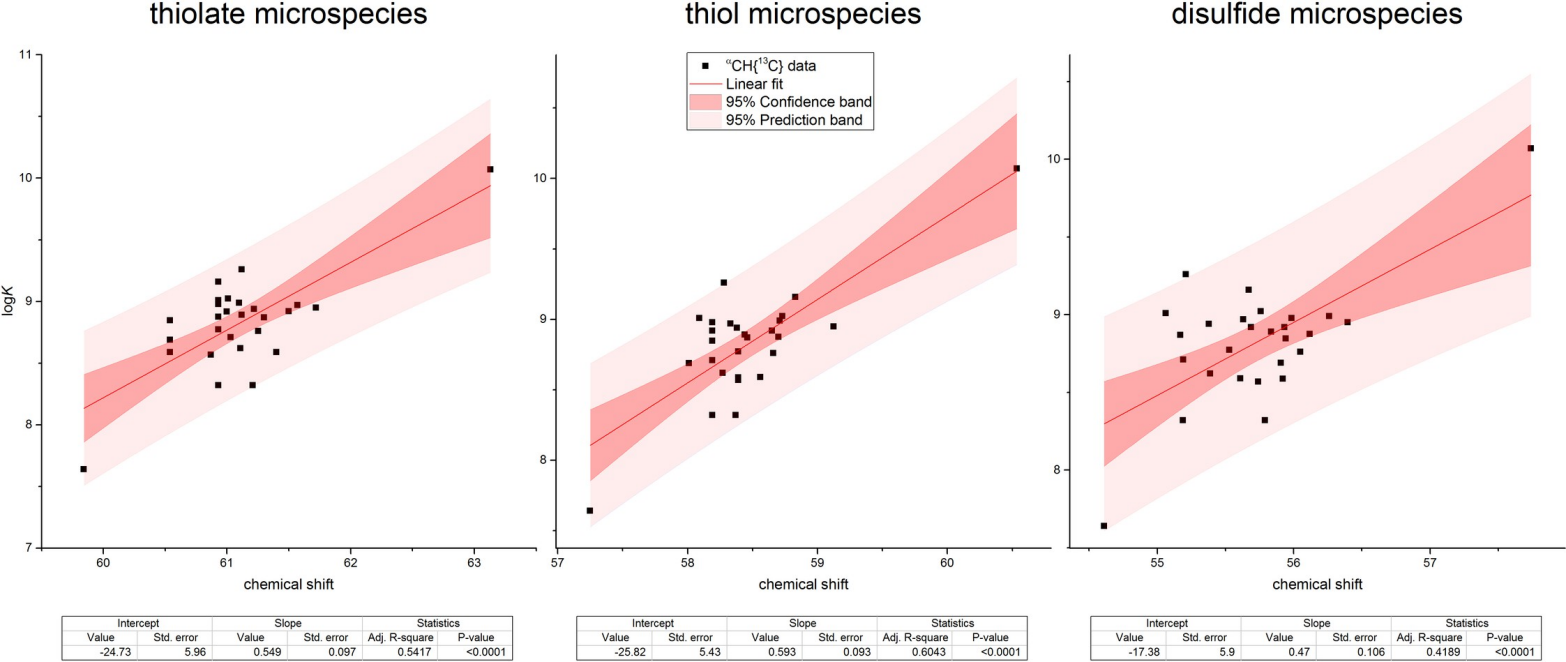
The univariate linear regression fits of the ^α^CH ^13^C data for all studied compounds together with 95% confidence and prediction bands.

In order to extend the validity of the multiple linear regression model obtained from cysteine and cystine date, we chose to include other cysteine-derivatives in the analysis; notably glutathione and other tripeptides meant to model the varying environments of mid-chain cysteine residues. We assumed that cysteine residues with neighboring amino acids of varying electronic effects (compounds **1**–**11**) would exhibit varying acid-base and NMR characteristics depending on their residue neighbor. Certain non-peptide cysteine derivatives with extremely electron withdrawing conjugates (compounds **12**–**15**) were also chosen in order to extend the log*K* spectrum in which data points could be acquired. The usual range of cysteine thiolate protonation constants is expected to fall between log*K* 8 and 10; however, since oxidoreductase enzymes must have reactive cysteine residues bearing unprotonated thiolate moieties for catalysis, there are indeed some instances in which the cysteine thiolate log*K* was found to be much lower than 7 (i.e. 3.5 or 4) [17–20]. Therefore, we hoped to extend the log*K* range of the linear model well below 7 by examining the compounds selected for further investigation, that are listed in Table 3. The species-specific protonation constants of glutathione and glutathione disulfide were imported from previous works [9, 14]. The microspecies notations of glutathione microspecies can be found in Fig 3. The thiolate protonation constants of the remaining compounds were determined with ^1^H NMR-pH titrations by plotting the ^1^H chemical shift of the cysteine ^α^CH vs pH. Non-linear regression analyses afforded the protonation constants using Eq (1). Based on the protonation constants compiled in Table 3, it is apparent that the originally anticipated lower thiolate log*K* values were not observed in the cysteine derivatives **12**–**15**. Tables 3–5 contain the NMR chemical shift data determined for these additional compounds as well. In Fig 4 the linear regression fits of only the best correlating ^α^CH ^13^C chemical shifts are shown for the entire data set.

**Table 3:** The species-specific chemical shifts determined for the additional compounds studied (beyond cysteine and cystine) bearing thiolate moiety. The uncertainty of determination for chemical shift on the ppm scale is 0.001 and 0.01 for ^1^H and ^13^C chemical shift values, respectively. The thiolate protonation constants of glutathione were determined previously in [14], while the protonation constants of the peptides were determined as described in the Materials and Methods section and are reported in logk ± standard deviation of the regression fit.

compound/thiolate species		thiolate log*k*	^β^CH_2_			^α^CH	
			*δ*^1^H		*δ*^13^C	*δ*^1^H	*δ*^13^C
GSH	GSH A	9.26±0.01	2.777	2.856	29.68	4.220	61.12
	GSH B	8.94±0.02	2.786	2.855	29.65	4.207	61.22
	GSH D	8.87±0.02	2.779	2.858	29.77	4.230	61.30
	GSH E	9.01±0.02	2.779	2.855	29.49	4.207	60.93
	GSH G	8.59±0.05	2.788	2.857	29.74	4.218	61.40
	GSH H	8.71±0.03	2.789	2.854	29.46	4.195	61.03
	GSH K	8.62±0.01	2.781	2.857	29.58	4.218	61.11
	GSH N	8.32±0.02	2.790	2.856	29.55	4.205	61.21
(**1**) alanylcysteinylalanine		8.98±0.04	2.745	2.930	29.49	4.126	60.93
(**2**) serylcysteinylserine		8.85±0.05	2.871	2.942	29.68	4.320	60.54
(**3**) valinylcysteinylvaline		8.97±0.07	2.711	2.923	29.51	4.190	61.57
(**4**) threonylcysteinylthreonine		8.89±0.03	2.808	2.964	29.68	4.287	61.12
(**5**) asparagylcysteinylasparagine		8.92±0.01	2.948	2.986	29.12	4.186	61.50
(**6**) acetylarginylcysteinylarginine amide		8.32±0.02	2.831	2.860	29.49	4.191	60.93
(**7**) acetylthreonylcysteinylthreonine amide		8.57±0.01	2.855	2.885	29.49	4.306	60.87
(**8**) acetylalanylcysteinylalanine amide		8.69±0.02	2.824	2.852	29.49	4.176	60.54
(**9**) acetylvalinylcysteinylvaline amide		8.88±0.03	2.782	2.844	29.49	4.238	60.93
(**10**) acetylasparagylcysteinylasparagine amide		8.77±0.02	2.756	2.845	29.48	4.196	60.93
(**11**) acetylserylcysteinylserine amide		8.59±0.006	2.870	2.903	29.49	4.319	60.54
(**12**) 4-methoxybenzoylcysteine amide		9.16±0.01	2.911	2.990	29.88	4.329	60.93
(**13**) 4-nitrobenzoylcysteine amide		8.99±0.02	2.928	3.015	29.90	4.380	61.10
(**14**) 4-dimethylaminobenzoylcysteine amide		8.92±0.01	2.928	2.991	29.80	4.326	61.00
(**15**) 3,5-bis(trifluoromethyl)benzoylcysteine amide		9.02±0.01	2.922	2.999	29.86	4.345	61.01

**Table 4:** The species-specific chemical shifts together with the thiolate-specific protonation shifts determined for the additional compounds studied (beyond cysteine and cystine) bearing thiol moiety. The uncertainty of determination for chemical shift on the ppm scale is 0.001 and 0.01 for ^1^H and ^13^C chemical shift values, respectively. Mean value and standard deviation of the protonation shifts (Δδ) are: 0.14±0.07, 0.08±0.05, -1.62±0.28, 0.37±0.05, -2.64±0.30 for the nuclei found in the table, respectively.

compound/thiol species		^β^CH_2_			^α^CH		^β^CH_2_			^α^CH	
		*δ*^1^H		*δ*^13^C	*δ*^1^H	*δ*^13^C	Δ*δ*^1^H		Δ*δ*^13^C	Δ*δ*^1^H	Δ*δ*^13^C
GSH	GSH C	2.919	2.975	28.34	4.580	58.28	*0*.*14*	*0*.*12*	*-1*.*3*	*0*.*36*	*-2*.*8*
	GSH F	2.929	2.974	28.31	4.568	58.38	*0*.*14*	*0*.*12*	*-1*.*3*	*0*.*36*	*-2*.*8*
	GSH I	2.921	2.977	28.43	4.591	58.46	*0*.*14*	*0*.*12*	*-1*.*3*	*0*.*36*	*-2*.*8*
	GSH J	2.921	2.973	28.15	4.568	58.09	*0*.*14*	*0*.*12*	*-1*.*3*	*0*.*36*	*-2*.*8*
	GSH L	2.930	2.976	28.40	4.578	58.56	*0*.*14*	*0*.*12*	*-1*.*3*	*0*.*36*	*-2*.*8*
	GSH M	2.931	2.972	28.12	4.555	58.19	*0*.*14*	*0*.*12*	*-1*.*3*	*0*.*36*	*-2*.*8*
	GSH O	2.923	2.975	28.24	4.578	58.27	*0*.*14*	*0*.*12*	*-1*.*3*	*0*.*36*	*-2*.*8*
	GSH P	2.933	2.974	28.21	4.566	58.37	*0*.*14*	*0*.*12*	*-1*.*3*	*0*.*36*	*-2*.*8*
(**1**)		2.927	2.990	28.12	4.547	58.19	*0*.*18*	*0*.*06*	*-1*.*4*	*0*.*42*	*-2*.*7*
(**2**)		2.968	3.026	28.12	4.680	58.19	*0*.*10*	*0*.*08*	*-1*.*6*	*0*.*36*	*-2*.*4*
(**3**)		2.896	2.977	27.97	4.658	58.33	*0*.*18*	*0*.*05*	*-1*.*5*	*0*.*47*	*-3*.*2*
(**4**)		2.967	3.035	28.12	4.727	58.44	*0*.*16*	*0*.*07*	*-1*.*6*	*0*.*44*	*-2*.*7*
(**5**)		2.970	3.028	27.93	4.610	58.19	*0*.*02*	*0*.*04*	*-1*.*2*	*0*.*42*	*-3*.*3*
(**6**)		2.936	2.973	27.73	4.494	58.19	*0*.*10*	*0*.*11*	*-1*.*8*	*0*.*30*	*-2*.*7*
(**7**)		2.972	2.983	27.93	4.636	58.39	*0*.*12*	*0*.*10*	*-1*.*6*	*0*.*33*	*-2*.*5*
(**8**)		2.925	2.962	27.93	4.497	58.01	*0*.*10*	*0*.*11*	*-1*.*6*	*0*.*32*	*-2*.*5*
(**9**)		2.888	2.935	27.85	4.570	58.70	*0*.*11*	*0*.*09*	*-1*.*6*	*0*.*33*	*-2*.*2*
(**10**)		2.818	2.896	27.73	4.525	58.39	*0*.*06*	*0*.*05*	*-1*.*8*	*0*.*33*	*-2*.*5*
(**11**)		2.965	2.996	27.73	4.613	58.39	*0*.*10*	*0*.*09*	*-1*.*8*	*0*.*29*	*-2*.*2*
(**12**)		3.018	3.098	28.10	4.672	58.83	*0*.*11*	*0*.*11*	*-1*.*8*	*0*.*34*	*-2*.*1*
(**13**)		3.037	3.119	27.80	4.872	58.71	*0*.*11*	*0*.*10*	*-2*.*1*	*0*.*49*	*-2*.*4*
(**14**)		3.014	3.092	27.90	4.660	58.65	*0*.*09*	*0*.*10*	*-1*.*9*	*0*.*33*	*-2*.*4*
(**15**)		3.023	3.103	27.93	4.734	58.73	*0*.*10*	*0*.*10*	*-1*.*9*	*0*.*39*	*-2*.*3*

**Table 5:** The species-specific chemical shifts determined for the additional compounds studied (beyond cysteine and cystine) bearing disulfide moiety. The uncertainty of determination for chemical shift on the ppm scale is 0.001 and 0.01 for ^1^H and ^13^C chemical shift values, respectively. The microspecies symbols for glutathione disulfide are not all depicted on Fig 3; labeling here is assumed to continue on the GSSG microspeciation scheme in alphabetical order, with labeling continuing after Z with AA, AB, and so on.

compound/disulfide species		^β^CH_2_			^α^CH	
		*δ*^1^H		*δ*^13^C	*δ*^1^H	*δ*^13^C
GSSG	GSSG A’	2.947	3.298	41.06	4.758	55.21
	GSSG E’	2.967	3.297	41.45	4.746	55.38
	GSSG J’	2.956	3.343	40.85	4.769	55.17
	GSSG M’	2.964	3.296	41.28	4.746	55.06
	GSSG X’	2.976	3.343	40.83	4.755	55.61
	GSSG AA’	2.957	3.292	41.24	4.732	55.19
	GSSG AF’	2.963	3.342	40.63	4.755	55.39
	GSSG AJ’	3.015	3.275	41.36	4.744	55.19
homodisulfide of (**1**)		3.146	3.178	42.41	4.300	55.98
homodisulfide of (**2**)		3.088	3.421	42.35	4.278	55.94
homodisulfide of (**3**)		3.207	3.400	42.68	4.261	55.63
homodisulfide of (**4**)		3.153	3.435	42.29	4.341	55.83
homodisulfide of (**5**)		2.989	3.185	42.36	4.300	55.69
homodisulfide of (**6**)		3.007	3.363	42.50	4.522	55.79
homodisulfide of (**7**)		2.946	3.150	41.91	4.677	55.74
homodisulfide of (**8**)		3.177	3.156	42.38	4.320	55.91
homodisulfide of (**9**)		3.088	3.257	42.26	4.299	56.12
homodisulfide of (**10**)		2.931	3.367	42.05	4.542	55.53
homodisulfide of (**11**)		2.962	3.083	42.42	4.628	55.92
homodisulfide of (**12**)		3.100	3.312	42.26	4.299	55.67
homodisulfide of (**13**)		2.988	3.351	42.27	4.281	56.26
homodisulfide of (**14**)		3.189	3.126	42.26	4.293	55.93
homodisulfide of (**15**)		3.089	3.257	42.41	4.285	55.76

## 4. Discussion

Cysteine is the most important thiol-bearing amino acid and the pivotal regulator of redox homeostasis and signaling. However, not all cysteine residues in proteins are likely to be oxidized, it will depend on the solvent accessibility, thiolate basicity and polarity of the nearby residues [7]. The analysis of the chemical shift data reveals a direct and inverse relationship between log*K* and ^13^C/^1^H chemical shifts, respectively. It was also observed that in terms of correlation, the ^1^H protonation shifts are considerably lower compared to the ^13^C counterparts. Furthermore, there are smaller differences between the reduced and oxidized species in terms of ^1^H chemical shifts as well. Contrarily, the ^13^C chemical shift data of the ^α^CH reveal the redox state of species as well the relevant physico-chemical properties. Corroborating this finding, in a previous study, Sharma and Rajarathnam already demonstrated that ^13^C NMR chemical shifts can clearly indicate disulfide bond structure and recognize the reduced and oxidized state of cysteine [21].

Regarding the protonation constant results of the cysteine-containing peptides (Table 3), it is interesting to observe that the presence of neighboring amino acid residues (even with highly electron withdrawing groups) do not influence the thiolate basicity, i.e. the neighboring residue on a cysteine has virtually no bearing on the acid-base/redox properties of the cysteine thiolate. This leads us the conclusion that the properties of a cysteine side-chain can only be perturbed via steric interactions in a peptide. The regression analysis presented in Fig 2 reveals a strong linear relationship between chemical shifts and thiolate basicities within the data of cysteine and cystine microspecies, whereas the correlation data from other compounds (Tables 3–5) only show adherence for the case of ^α^CH ^13^C. The data from the ^α^CH ^13^C nucleus of the studied peptides show the best conformity to the linear correlation of cysteine data; therefore this nucleus is the best possible option to estimate thiolate properties from NMR data. The linear regression fits on this nucleus alone are shown for all studied compounds in Fig 4. The reason why this alpha carbon is the best indicator of thiolate characteristics is probably due to the position of the ^α^CH carbon relative to the sulfur atom as they are optimally connected via two covalent bonds to each other; the optimal covalent distance for NMR reporter nuclei. On the contrary, however, the ^α^CH ^1^H chemical shifts of the cysteine are presumably perturbed more by the protonation state of neighboring moieties or the presence of a peptide bond, disqualifying this nucleus to be an indicator of the properties of the sulfur atom. The ^β^CH_2_ nuclei also show this phenomenon and have a weaker correlation with log*K* altogether. These observations hold for the regression analysis of the thiolate bearing species as well as that of the thiol bearing and the concomitant disulfide bearing species.

It is often assumed that chemical shifts are highly susceptible to changes in the microenvironment of the NMR active nuclei. Moreover, the correlation from cysteine chemical shifts, log*K*, and redox potentials could bring better knowledge about the chemistry and the biological function of its oxidation [21]. Through the accrued chemical shift data set and regression analysis, it is also possible to estimate thiolate basicity/thiol acidity and the concomitant standard redox potential.

Nevertheless, the obvious limitation of this method is the window of the regression analysis. Since the species-specific thiolate basicities observed in cysteine are limited to a certain window, and the further analysis of derivative compounds did not extend this range, in order to further the scale of the regression more measurements on larger peptides are needed. We performed a thorough literature search for reported thiolate log*K* values in the PKAD Database [22] and the corresponding chemical shift values for the cysteine residue using the Biological Magnetic Resonance Data Bank (BMRM) (http://www.bmrb.wisc.edu). Unfortunately, the literature review produced only a handful of data that seems to be unreliable to incorporate into the model. Our research group is currently investigating larger peptides as we hope that the determination of species-specific chemical shifts of added peptides will extend the regression model for better utility.

## 5. Conclusion

It was possible to confirm a strong linear relationship within the cysteine microspecies for the chemical shift data vs thiolate, specifically for the ^α^CH ^13^C. The next step in improving this model is to analyze peptides with lower thiolate basicity and extend the correlation that can be used on larger proteins in order to estimate acid-base and redox character of cysteine residues using their NMR chemical shifts.

## Supporting information

S1 Fig NMR spectra of the studied peptides. (DOCX)

S2 Fig ^1^H NMR chemical shift of the alpha CH vs pH profiles of the peptides (see Materials and Methods for measurement details). (DOCX)

S1 Graphical abstract (TIF)
