# Supplementary material for: Close correlation between thiolate basicity and certain NMR parameters in cysteine and cystine microspecies
Source: PLoS One. 2022 Mar 11;17(3):e0264866. doi: 10.1371/journal.pone.0264866 (PMC8916652; doi:10.1371/journal.pone.0264866)
Supplement: S1 Fig — (DOCX) [file pone.0264866.s001.docx]

**S1 Fig**

Close correlation between thiolate basicity and certain NMR parameters in cysteine and cystine microspecies

**AUTHORS**

Juliana Ferreira de Santana^1^, Arash Mirzahosseini^1,2^, Beáta Mándity^3^, Dóra Bogdán^3,4^, István Mándity^3,4^, Béla Noszál^1,2^*

*^1^Department of Pharmaceutical Chemistry, Semmelweis University, Budapest, Hungary*

*^2^Research Group of Drugs of Abuse and Doping Agents, Hungarian Academy of Sciences, Budapest, Hungary*

*^3^MTA TTK Lendület Artificial Transporter Research Group, Institute of Materials and Environmental Chemistry, Research Center for Natural Sciences, Hungarian Academy of Sciences, Budapest, Hungary*

*^4^Department of Organic Chemistry, Semmelweis University, Budapest, Hungary*

**S1 Fig. NMR spectra of the studied peptides**

(1) alanylcysteinylalanine

(2) serylcysteinylserine

(3) valinylcysteinylvaline

(4) threonylcysteinylthreonine

(5) asparagylcysteinylasparagine

(6) acetylarginylcysteinylarginine amide

(7) acetylthreonylcysteinylthreonine amide

(8) acetylalanylcysteinylalanine amide

(9) acetylvalinylcysteinylvaline amide

(10) acetylasparagylcysteinylasparagine amide

(11) acetylserylcysteinylserine amide

(12) 4-methoxybenzoylcysteine amide

(13) 4-nitrobenzoylcysteine amide

(14) 4-dimethylaminobenzoylcysteine amide

(15) 3,5-bis(trifluoromethyl)benzoylcysteine amid
