# Supplementary material for: Close correlation between thiolate basicity and certain NMR parameters in cysteine and cystine microspecies
Source: PLoS One. 2022 Mar 11;17(3):e0264866. doi: 10.1371/journal.pone.0264866 (PMC8916652; doi:10.1371/journal.pone.0264866)

**S2 Fig**

Close correlation between thiolate basicity and certain NMR parameters in cysteine and cystine microspecies

**AUTHORS**

Juliana Ferreira de Santana^1^, Arash Mirzahosseini^1,2^, Beáta Mándity^3^, Dóra Bogdán^3,4^, István Mándity^3,4^, Béla Noszál^1,2^*

*^1^Department of Pharmaceutical Chemistry, Semmelweis University, Budapest, Hungary*

*^2^Research Group of Drugs of Abuse and Doping Agents, Hungarian Academy of Sciences, Budapest, Hungary*

*^3^MTA TTK Lendület Artificial Transporter Research Group, Institute of Materials and Environmental Chemistry, Research Center for Natural Sciences, Hungarian Academy of Sciences, Budapest, Hungary*

*^4^Department of Organic Chemistry, Semmelweis University, Budapest, Hungary*

**S2 Fig. ^1^H NMR chemical shift of the alpha CH vs pH profiles of the peptides (see Materials and Methods for measurement details)**

(1) alanylcysteinylalanine


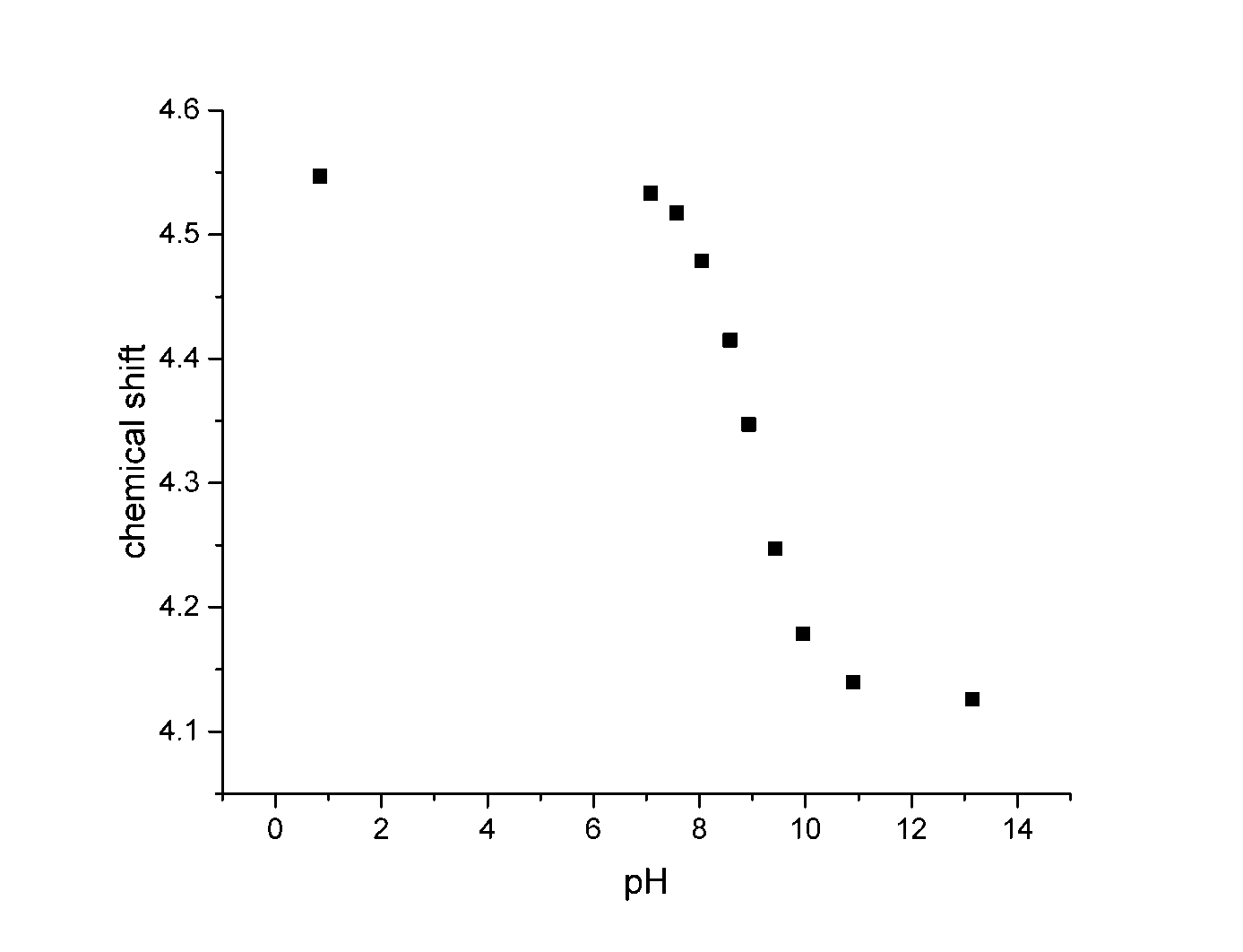


(2) serylcysteinylserine


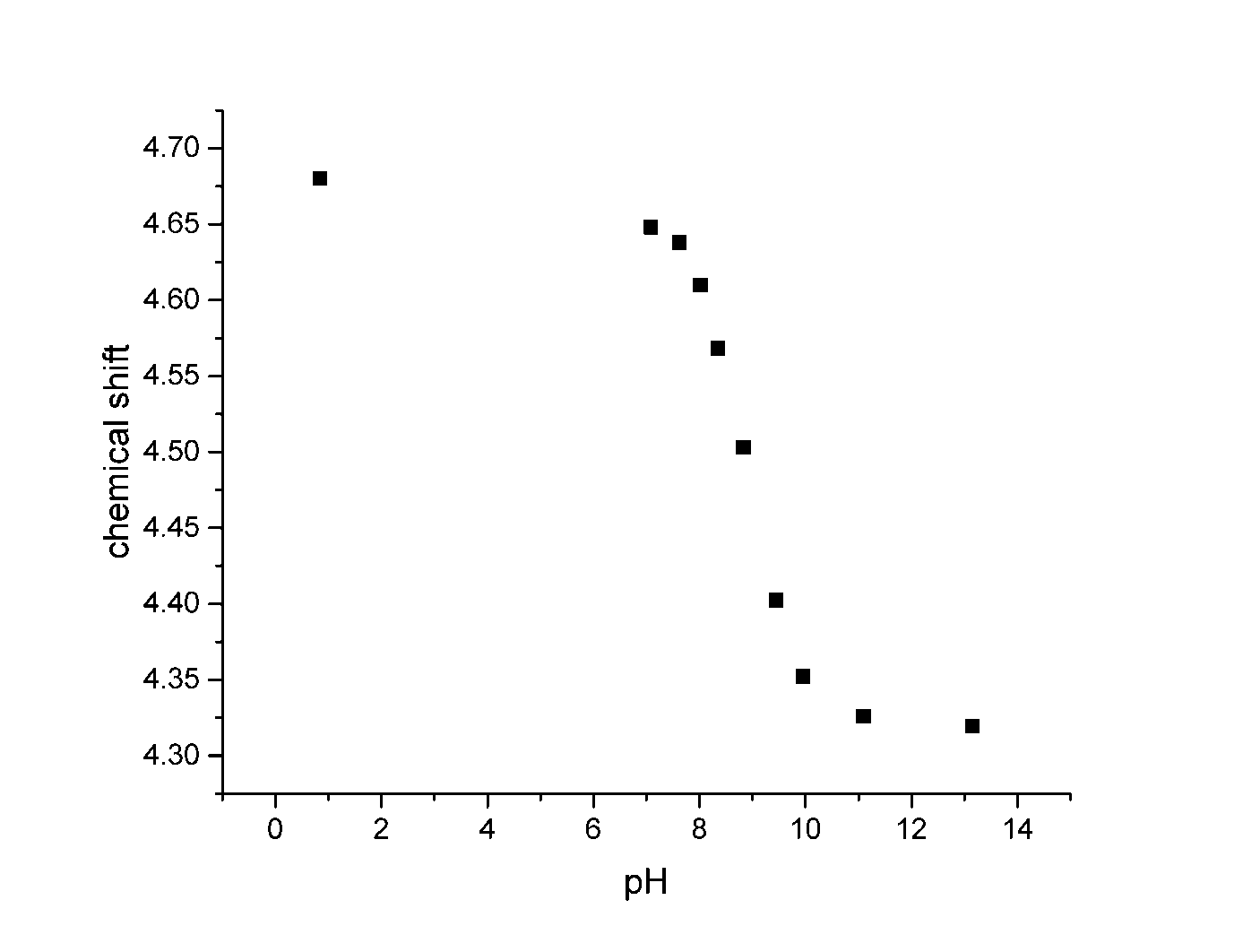
(3) valinylcysteinylvaline


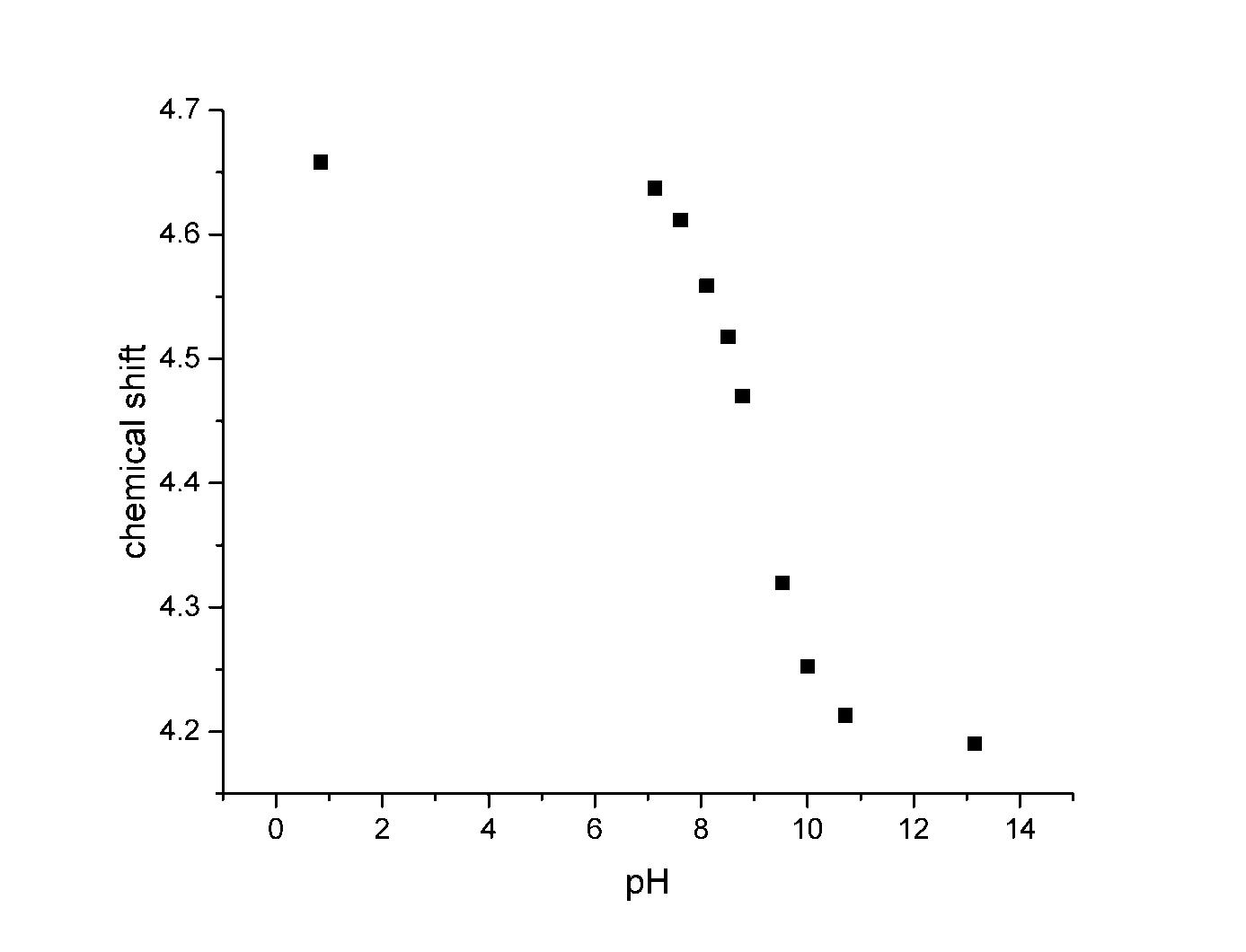


(4) threonylcysteinylthreonine


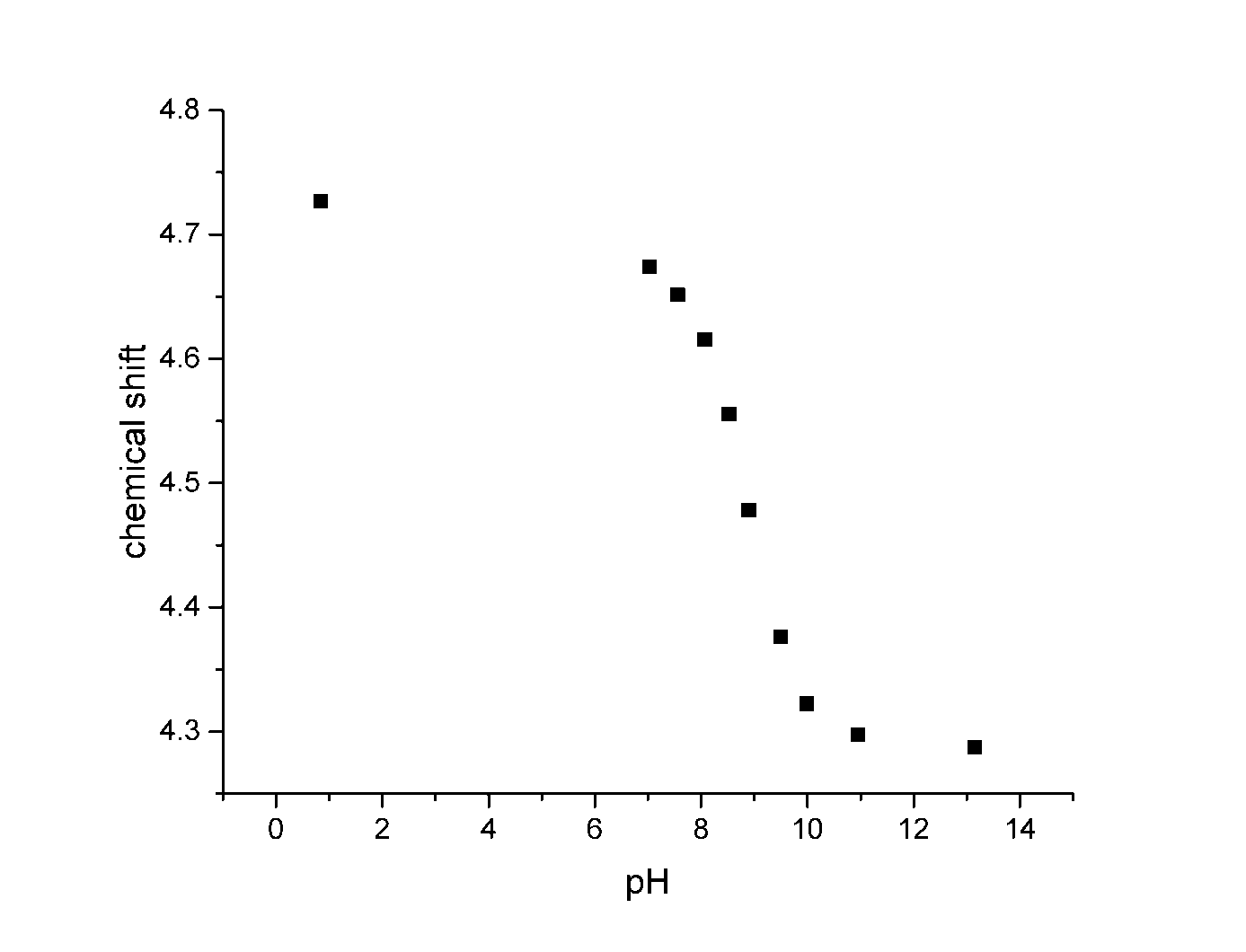
(5) asparagylcysteinylasparagine


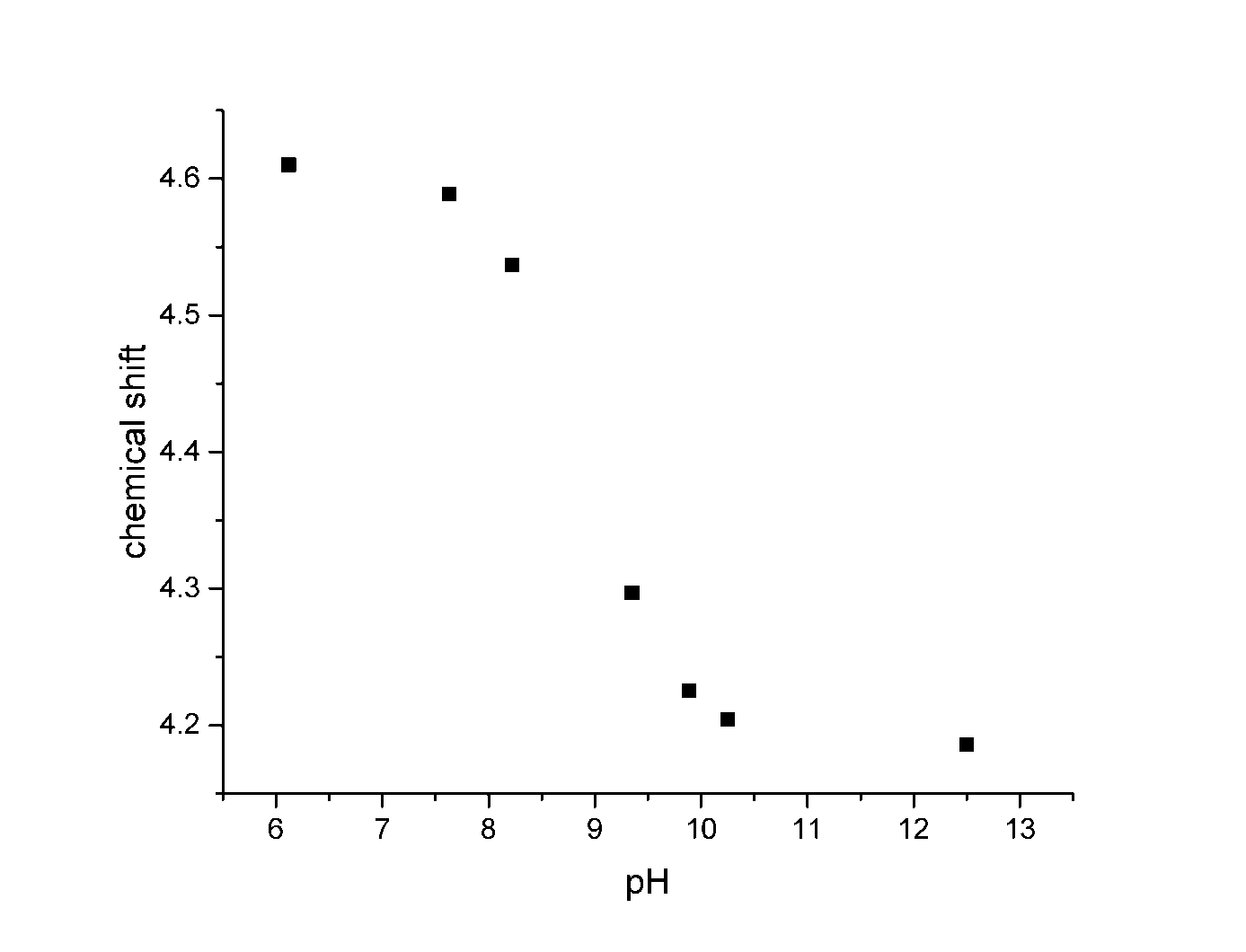


(6) acetylarginylcysteinylarginine amide


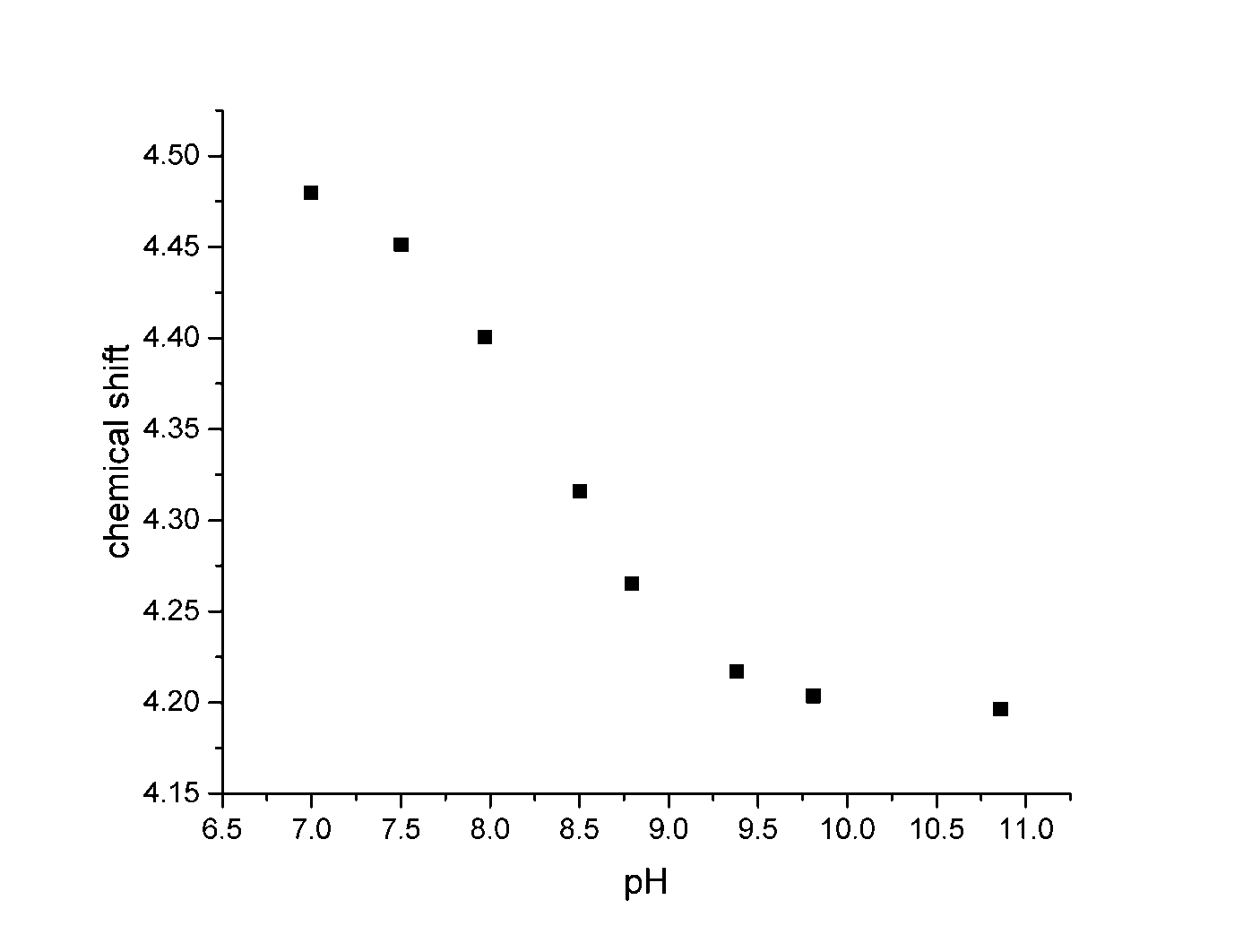
(7) acetylthreonylcysteinylthreonine amide


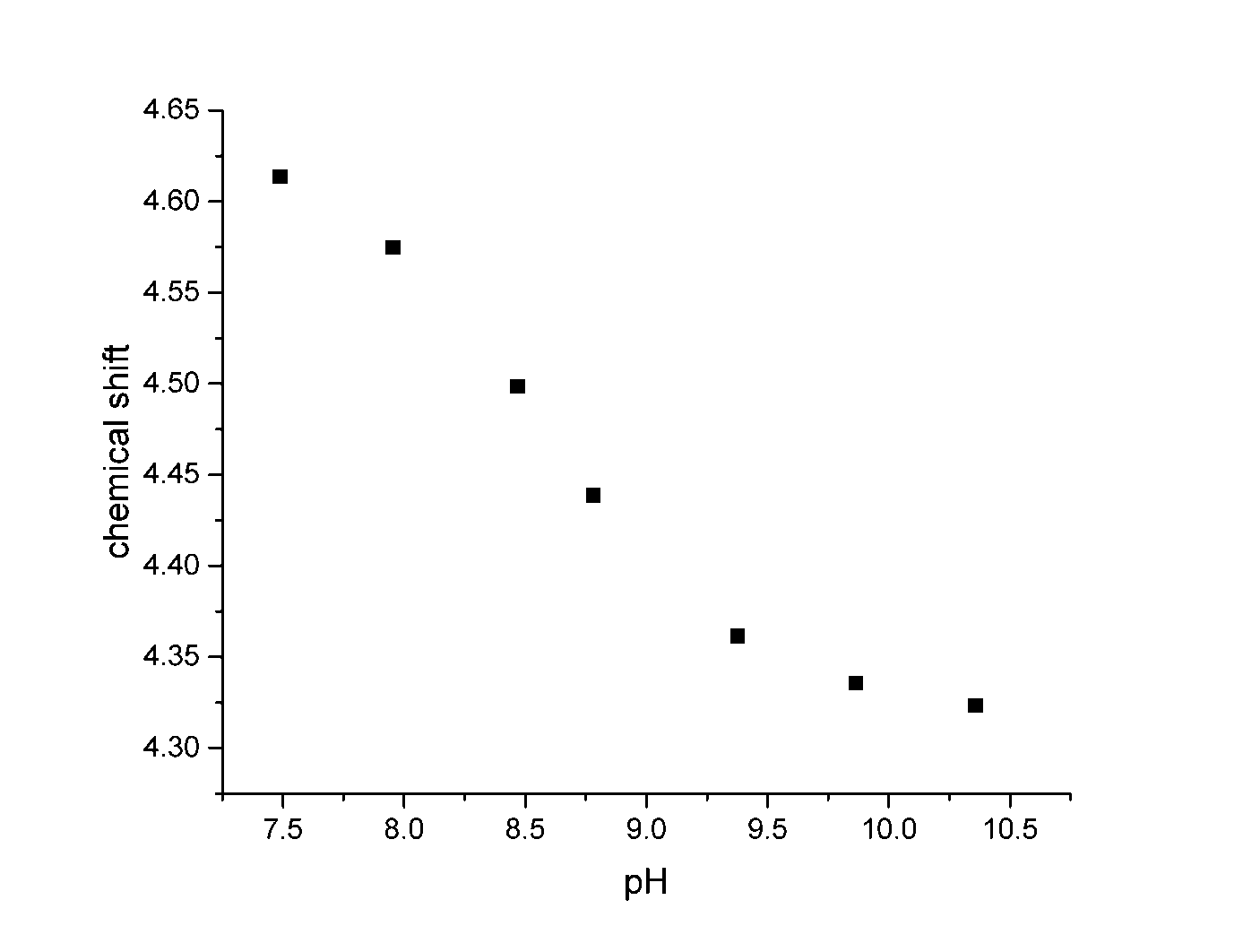


(8) acetylalanylcysteinylalanine amide


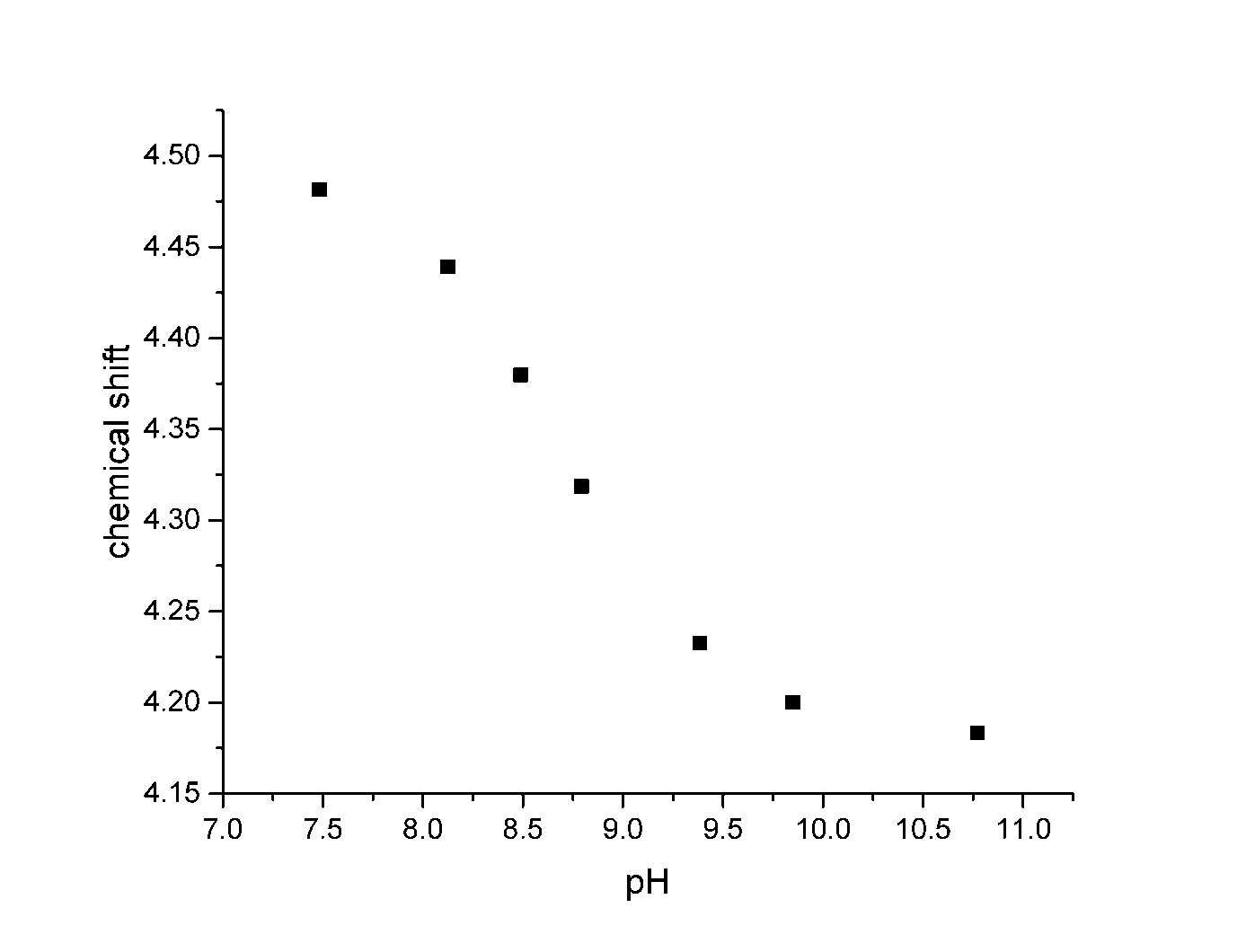
(9) acetylvalinylcysteinylvaline amide


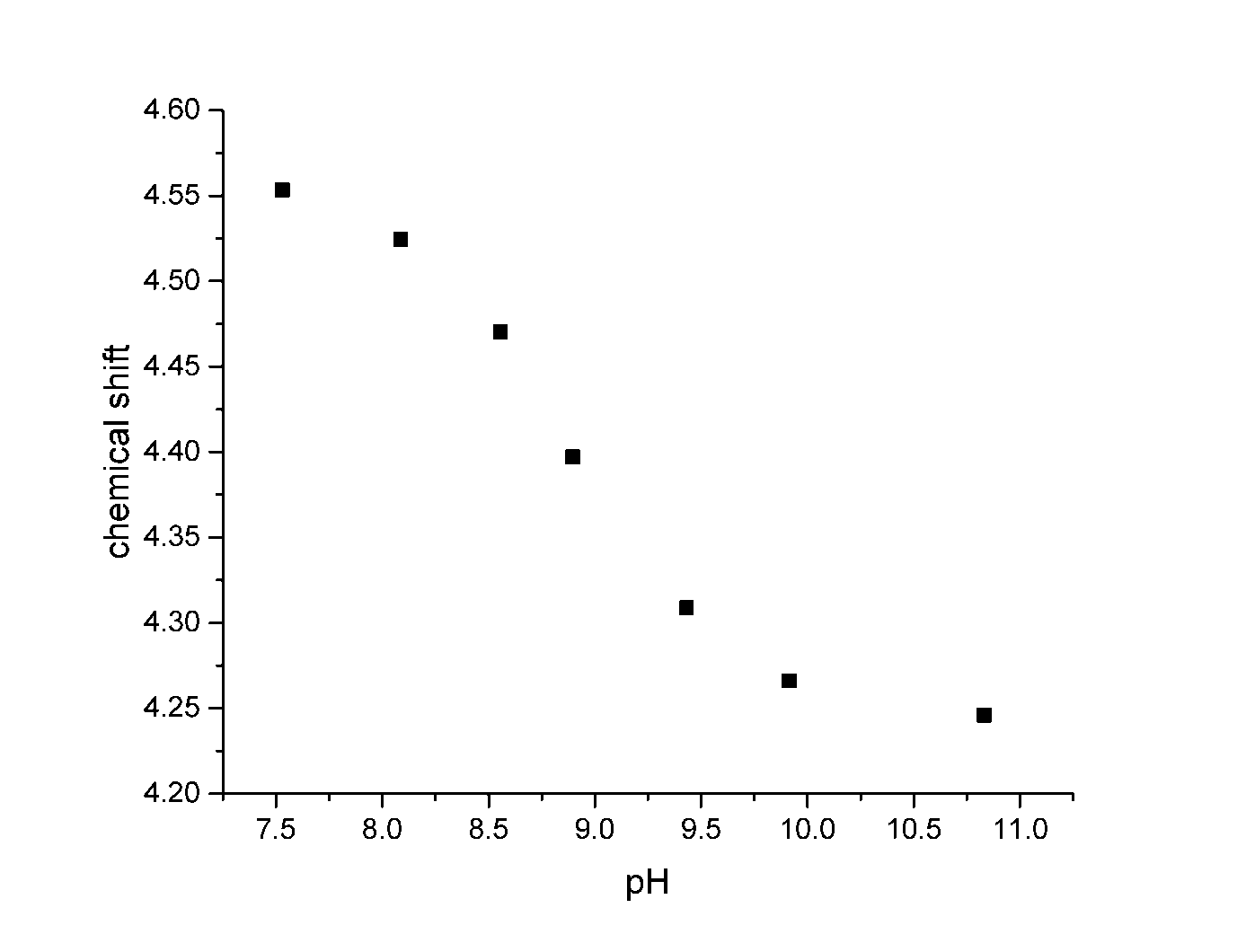


(10) acetylasparagylcysteinylasparagine amide


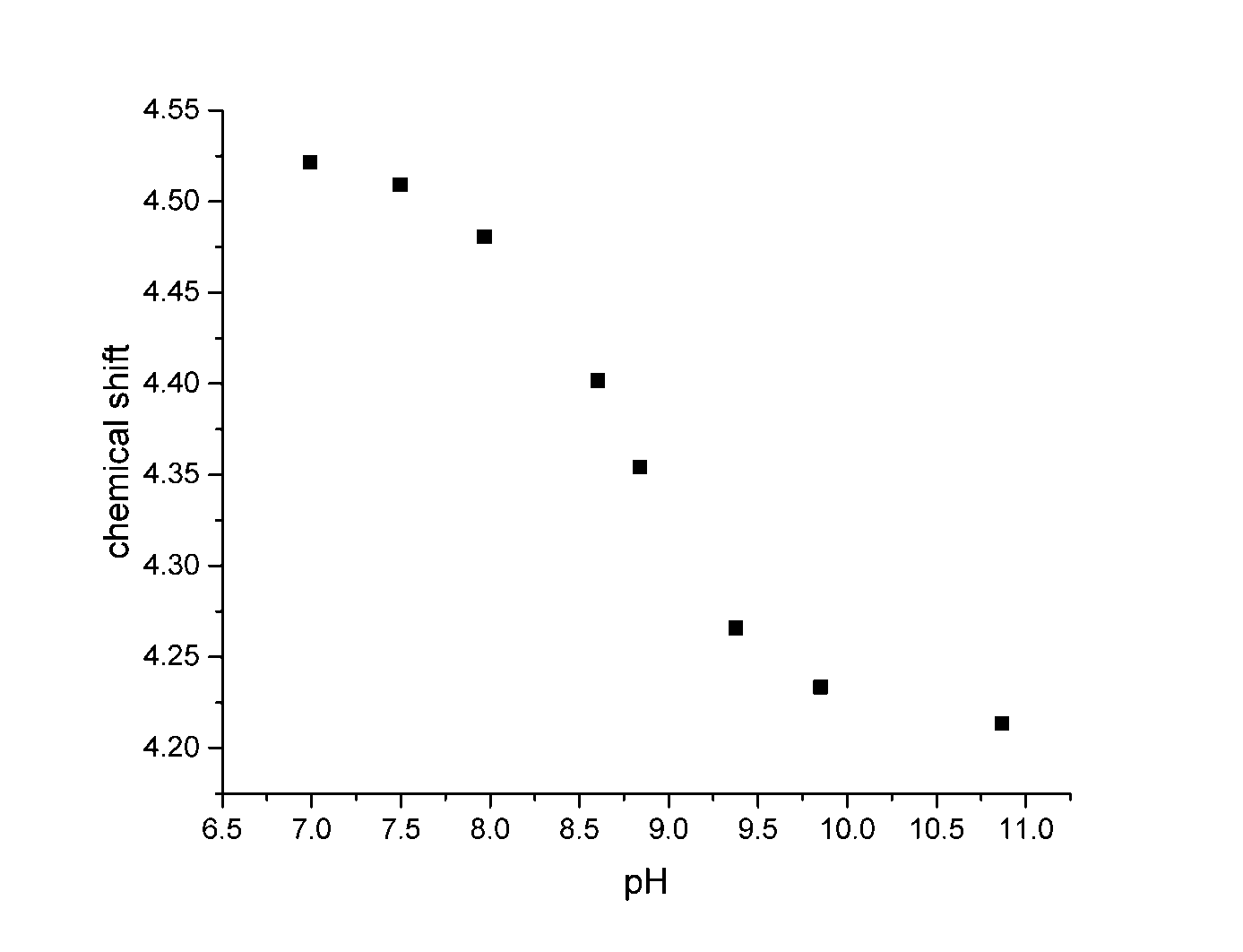
(11) acetylserylcysteinylserine amide


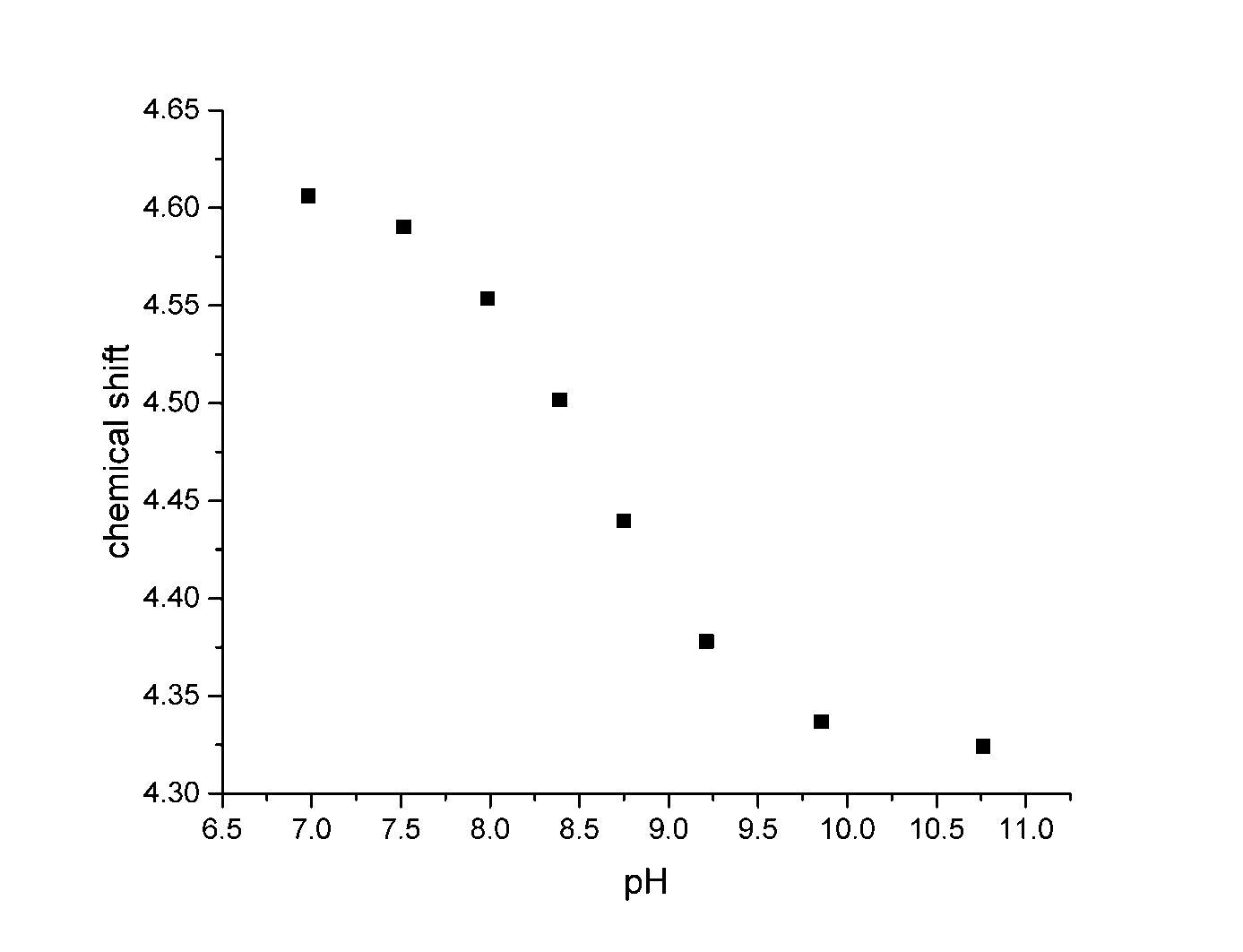


(12) 4-methoxybenzoylcysteine amide


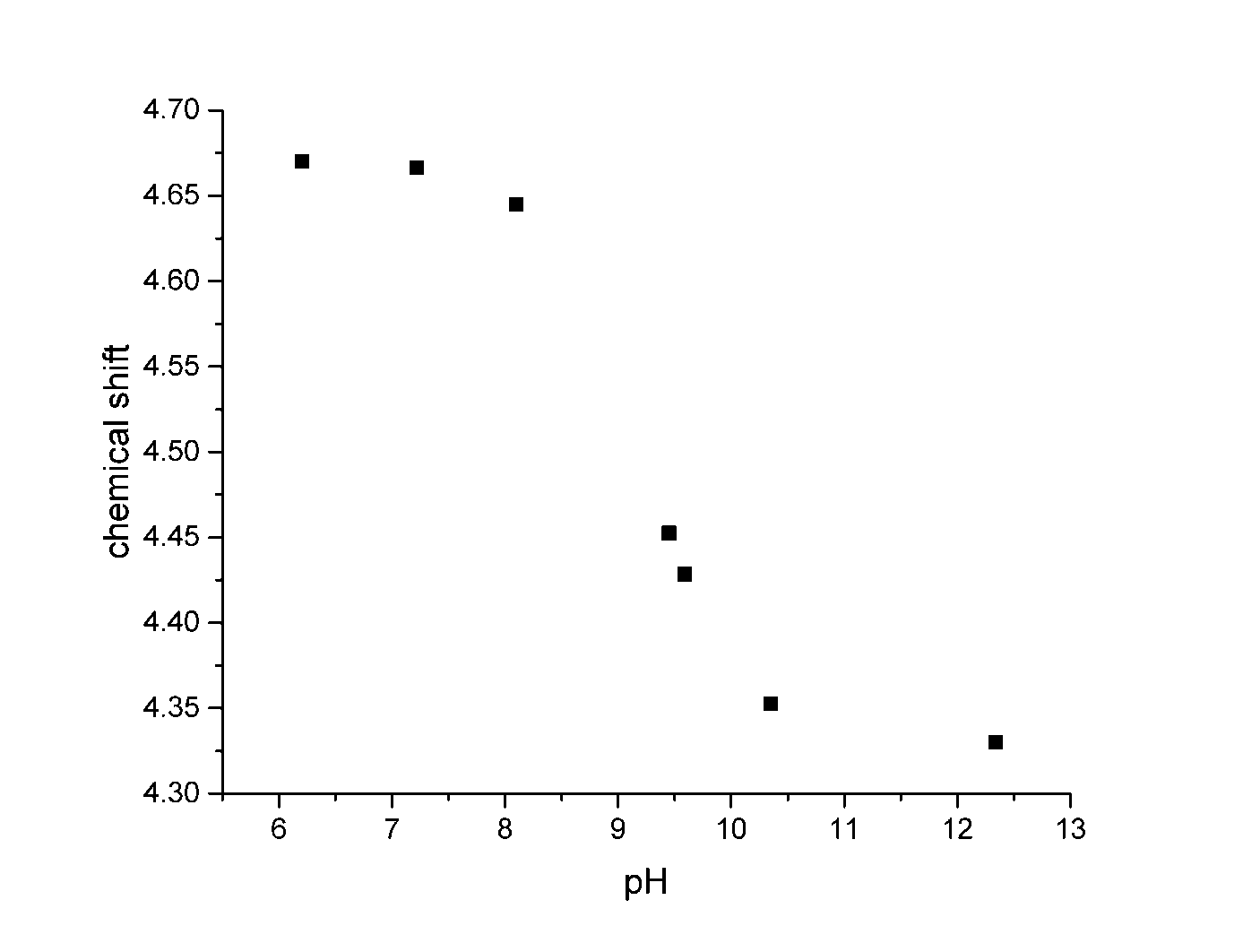
(13) 4-nitrobenzoylcysteine amide


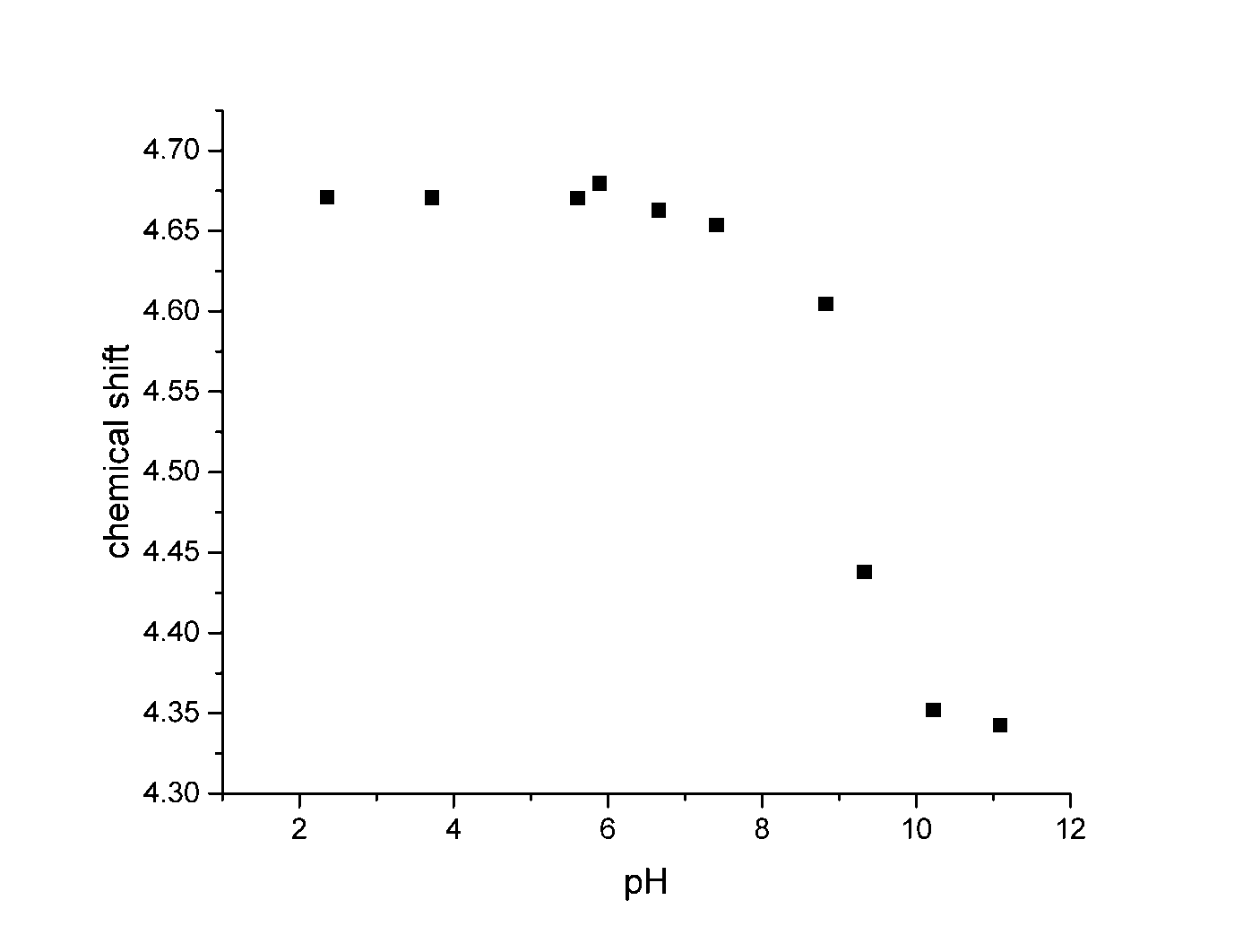


(14) 4-dimethylaminobenzoylcysteine amide


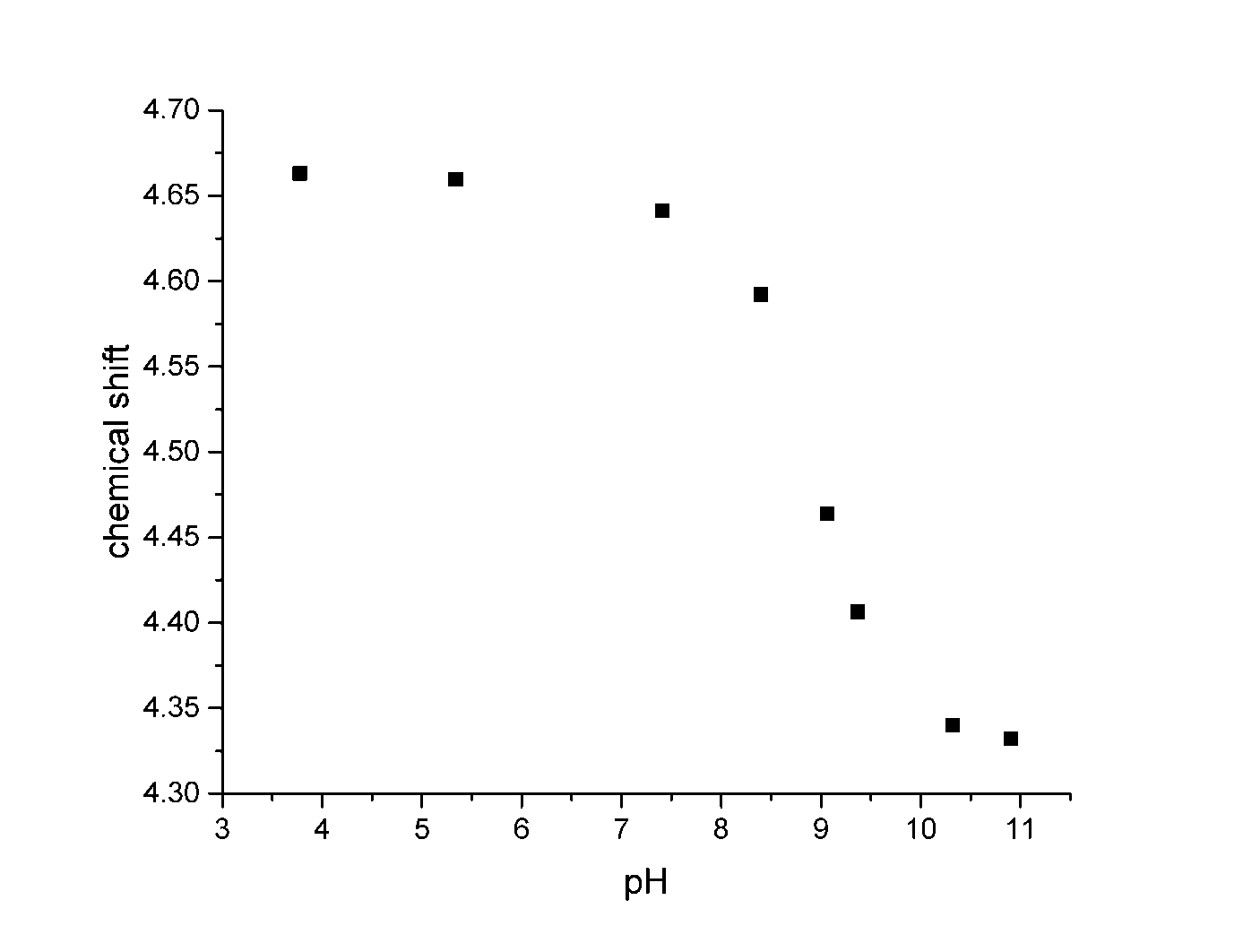
(15) 3,5-bis(trifluoromethyl)benzoylcysteine amide


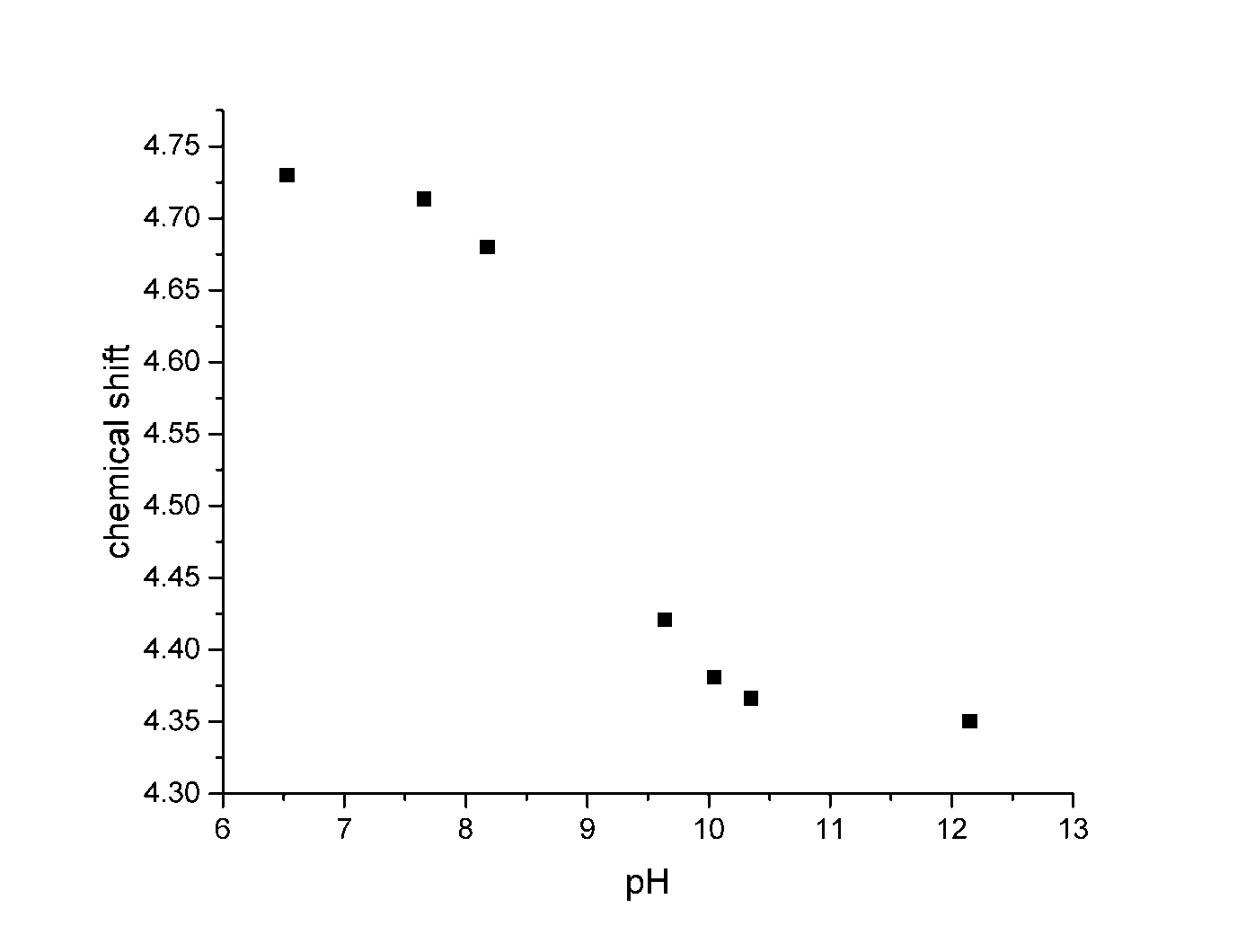

Supplement: S2 Fig — (DOCX) [file pone.0264866.s002.docx]
