# Supplementary figures and images for: Close correlation between thiolate basicity and certain NMR parameters in cysteine and cystine microspecies
Source: PLoS One. 2022 Mar 11;17(3):e0264866. doi: 10.1371/journal.pone.0264866 (PMC8916652; doi:10.1371/journal.pone.0264866)

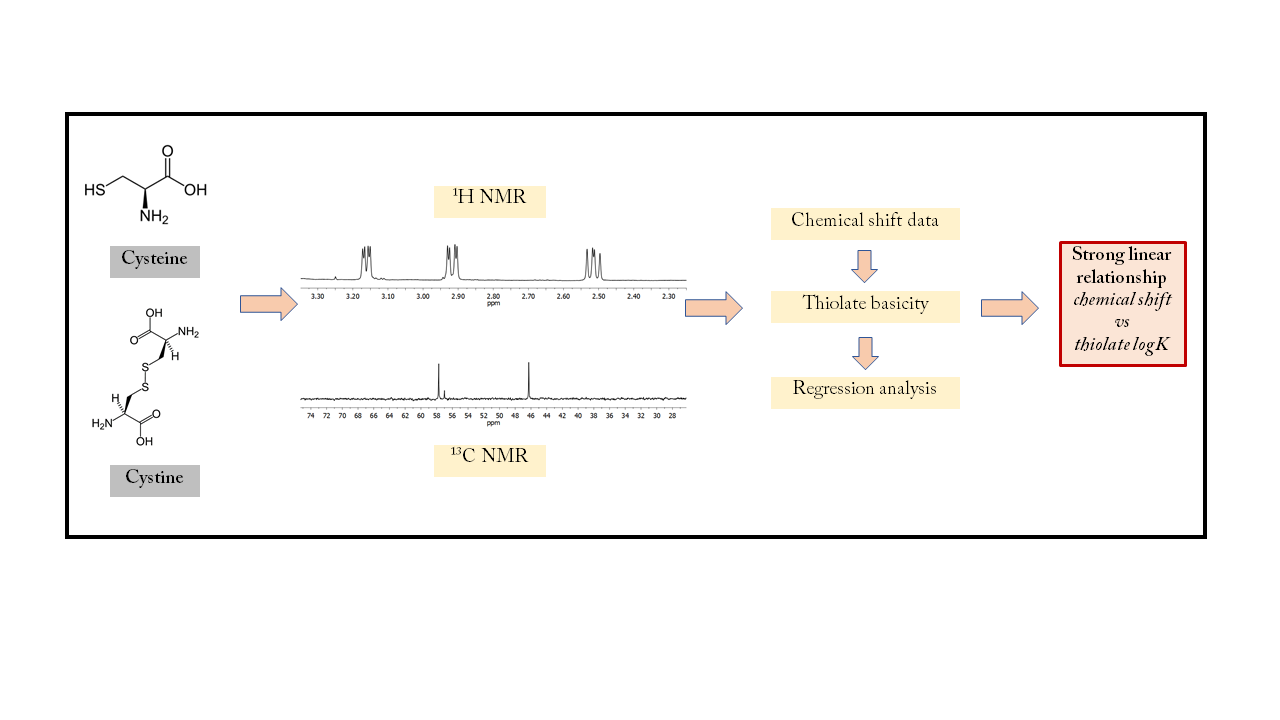

Supplement: S1 Graphical abstract — (TIF) [file pone.0264866.s003.tif]
